# An ultraprocessive, accurate reverse transcriptase encoded by a metazoan group II intron

**DOI:** 10.1261/rna.063479.117

**Published:** 2018-02

**Authors:** Chen Zhao, Fei Liu, Anna Marie Pyle

**Affiliations:** 1 Department of Molecular Biophysics and Biochemistry, Yale University, New Haven, Connecticut 06520, USA; 2 Department of Molecular, Cellular, and Developmental Biology, Yale University, New Haven, Connecticut 06520, USA; 3 Howard Hughes Medical Institute, Chevy Chase, Maryland 20815, USA; 4 Department of Chemistry, Yale University, New Haven, Connecticut 06520, USA

**Keywords:** group II intron, maturase, intron-encoded protein, reverse transcriptase, processivity, RNA-seq

## Abstract

Group II introns and non-LTR retrotransposons encode a phylogenetically related family of highly processive reverse transcriptases (RTs) that are essential for mobility and persistence of these retroelements. Recent crystallographic studies on members of this RT family have revealed that they are structurally distinct from the retroviral RTs that are typically used in biotechnology. However, quantitative, structure-guided analysis of processivity, efficiency, and accuracy of this alternate RT family has been lacking. Here, we characterize the processivity of a group II intron maturase RT from *Eubacterium rectale* (*E.r*.), for which high-resolution structural information is available. We find that the *E.r.* maturase RT (MarathonRT) efficiently copies transcripts at least 10 kb in length and displays superior intrinsic RT processivity compared to commercial enzymes such as Superscript IV (SSIV). The elevated processivity of MarathonRT is at least partly mediated by a loop structure in the finger subdomain that acts as a steric guard (the α-loop). Additionally, we find that a positively charged secondary RNA binding site on the surface of the RT diminishes the primer utilization efficiency of the enzyme, and that reengineering of this surface enhances capabilities of the MarathonRT. Finally, using single-molecule sequencing, we show that the error frequency of MarathonRT is comparable to that of other high-performance RTs, such as SSIV, which were tested in parallel. Our results provide a structural framework for understanding the enhanced processivity of retroelement RTs, and they demonstrate the potential for engineering a powerful new generation of RT tools for application in biotechnology and research.

## INTRODUCTION

Long RNA molecules control numerous aspects of gene expression, such as mRNAs, regulatory RNAs, viral genomes, components of the machinery for translation, RNA processing, and many other processes (Mercer et al. 2009; Kung et al. 2013). Unfortunately, our current understanding of the abundance, sequence, and structure of RNAs, and particularly long RNAs (>200 nt), is limited by the low processivity of the reverse transcriptase enzymes (RTs) that are used to copy RNA molecules into DNA products, or cDNAs, which are often subsequently amplified by PCR (RT-PCR). There are many negative consequences of this limitation, which include the following: (i) During analysis of transcriptome-wide gene expression, low RT processivity biases read coverage and transcript quantification, which is particularly severe in single-cell transcriptome profiling experiments (Archer et al. 2016). (ii) When using RNA structure probing methods such as SHAPE (Wilkinson et al. 2006; Spitale et al. 2015), low RT processivity results in a high background signal that can obscure results. (iii) Low RT processivity obstructs development of end-to-end long-read sequencing methods such as nanopore sequencing (Bolisetty et al. 2015) and SMRT sequencing (Pan et al. 2008). (iv) The short reads typical of conventional RTs limit the development of single-molecule direct RNA sequencing using the PacBio platform, in contrast to similar applications for DNA sequencing (SMRT) that are gaining popularity (Chaisson et al. 2015). To date, direct RNA sequencing has focused on the use of short reads (<56 nt) (Ozsolak et al. 2009; Vilfan et al. 2013), or it uses nanopore technology that has a relatively high error rate (Laver et al. 2015). (v) Conventional RTs have limited utility on highly structured or post-transcriptionally modified RNA. Long-read RNA sequencing methods are needed to unambiguously characterize heterogeneous populations of long RNA molecules, such as splice variants, viral quasi-species, and RNAs containing different modification, editing, or mutation sites, but this is not possible with current RT technology. Given these shortcomings, improved RT enzymes would have a transformative impact on RNA science.

**FIGURE 1.**
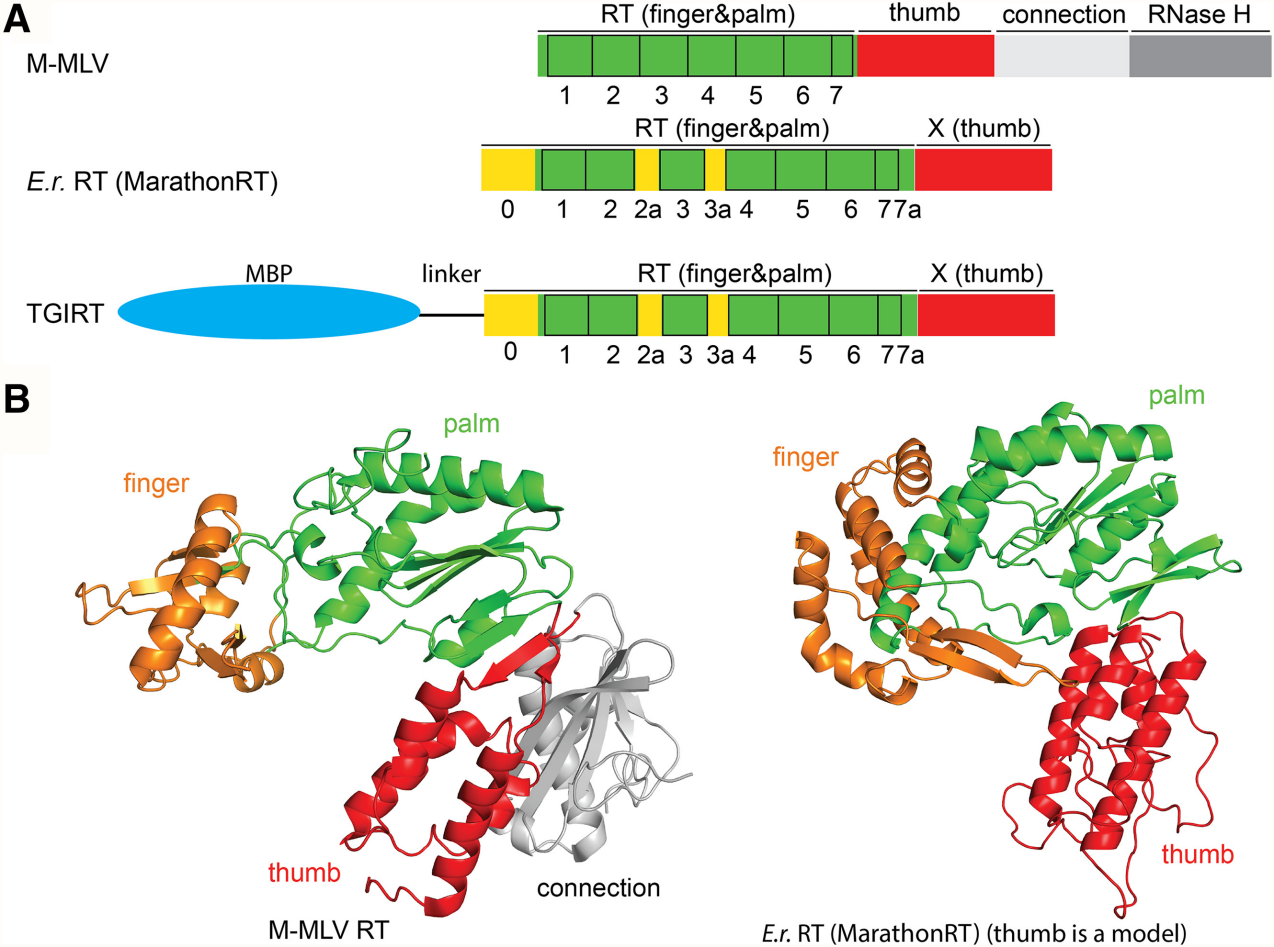
Sequence and structure of group II intron maturases. (*A*) Comparison of domain organization for different reverse transcriptase (RT) enzymes. The name of individual domains is labeled at the *top* of each sketch, whereas the seven conserved motifs within the RT (motifs 1–7, green) are indicated *below*. The N-terminal extension (0) and insertions between the conserved sequence blocks (motifs 2a, 3a, and 7a, yellow) are observed in group II intron maturases but not in retroviral RTs. (M-MLV) Moloney murine leukemia virus RT (71 kDa). (*E.r.*) Encoded by a group II intron from *Eubacterium rectale* (Eu.re.I2) and referred to as MarathonRT (47 kDa). (TGIRT) A commercial group II intron RT (InGex, LLC) derived from *Geobacillus stearothermophilus* stabilized by MBP (maltose binding protein, shown as blue ellipse). The molecular weight of MBP is ∼43 kDa, and the molecular weight of the RT is ∼48 kDa. (*B*) Three-dimensional structure of M-MLV RT (PDB ID: 4MH8) (Das and Georgiadis 2004) and group II intron maturases from *E.r.* (MarathonRT). The PDB ID for *E.r.* maturase RT domain is 5HHL, and the model for X domain (thumb) in *E.r.* maturase was created as a threading model by I-TASSER (Yang et al. 2015) based on the thumb subdomain of LtrA (PDB ID: 5G2Y).

The most well-studied and commonly utilized RTs derive from retroviruses (such as the Superscript series, which originated from the M-MLV [Moloney Murine Leukemia Virus] RT). However, RTs can be classified into several families based on sequence and structural homology (Xiong and Eickbush 1990; Zhao and Pyle 2016). A second family of RTs, distinct in sequence and domain organization, is found in non-long-terminal-repeat (non-LTR) retrotransposons (Xiong and Eickbush 1990) and within the intron-encoded proteins of group II introns (Kennell et al. 1993; Moran et al. 1994; Zimmerly et al. 1995; Matsuura et al. 1997). This class of RTs, known as maturase RTs, contains an N-terminal extension (RT0) and a specific set of insertions between the seven conserved sequence motifs that are found in all RTs (RT1–7) (Fig. 1A; Blocker et al. 2005; Zhao and Pyle 2017). The RT domain contains the typical finger and palm subdomains, includes the catalytic center, and mediates polymerase fidelity and processivity (Fig. 1A; Zimmerly et al. 2001; Blocker et al. 2005; Zhao and Pyle 2017). The C-terminal region of maturase RTs, known as the “X domain,” is analogous to a polymerase thumb, and it makes important contributions to polymerase processivity (Fig. 1A; Zimmerly et al. 2001; Blocker et al. 2005; Zhao and Pyle 2016, 2017). In vivo, each maturase RT forms a specific complex with its parent intron RNA, resulting in a stable RNP complex that carries out reverse-transcription during the course of retrotransposition (Saldanha et al. 1999; Qu et al. 2016; Zhao and Pyle 2016, 2017).

Maturase RT enzymes tend to be highly processive, as this is required for successful copying of the large, highly structured group II intron RNA and for successful propagation of group II introns within their hosts (Mohr et al. 2013; Lambowitz and Belfort 2015). Many studies have noted the unusually high processivity of group II intron maturases (Fig. 1A; Mohr et al. 2013) and related non-LTR retrotransposon RTs (Bibillo and Eickbush 2002; Cost et al. 2002; Piskareva and Schmatchenko 2006). In particular, a thermally stable group II intron maturase (known as TGIRT) has been successfully used for cDNA library construction (Mohr et al. 2013; Enyeart et al. 2014; Zheng et al. 2015; Nottingham et al. 2016; Qin et al. 2016; Zubradt et al. 2017). Despite these advances in utilizing new RT families, lack of structural and mechanistic information and limited efforts at optimization have hindered their widespread adoption in biotechnology.

We recently discovered a new reverse-transcriptase during the course of our structural investigations on mechanisms of group II intron splicing and retrotransposition. As our original goal was to obtain high-resolution structural data, we used bioinformatic methods to discover a group II intron maturase RT with improved physical properties such as enhanced solubility, stable folding, monodispersity, and good catalytic activity (Zhao and Pyle 2016). We succeeded in identifying a set of small maturase RT enzymes from metazoan bacteria that met these criteria, and we solved their structures to exceptionally high resolution (1.2 Å and 2.1 Å). This provided first-in-class structures of the distinct family of RT enzymes that are found in group II introns and non-LTR retrotransposons (Zhao and Pyle 2016). These structures set the stage for structure–function analysis on this promising family of RT enzymes, which displayed preliminary RT activity that was particularly robust (Zhao and Pyle 2016).

Here we characterize the enzymatic properties of this polymerase subfamily, focusing on the RT from bacterium *Eubacterium rectale* (*E.r.*) (Zhao and Pyle 2016). Attributes such as processivity, error frequency, and other parameters are examined and compared with values obtained in parallel on other RT enzymes such as TGIRT and Superscript IV (SSIV) (Fig. 1A). We find that the *E.r.* RT (henceforth called MarathonRT) displays extraordinary levels of processivity, even under conditions of excess template RNA, and that error frequency is comparable to other high-performance RTs. Subsequent structure–function analysis on the RT reveals the physical basis for superior RT processivity of group II intron maturases and related non-LTR RTs. Using data from the crystal structures of the *E.r.* RT domain, we designed mutations that improve the properties of the RT, thereby demonstrating that this family of RTs can be further optimized and engineered to create a new generation of powerful enzyme tools for meeting the needs of cutting-edge RNA science.

## RESULTS

### Efficient copying of a highly structured viral genome

**FIGURE 2.**
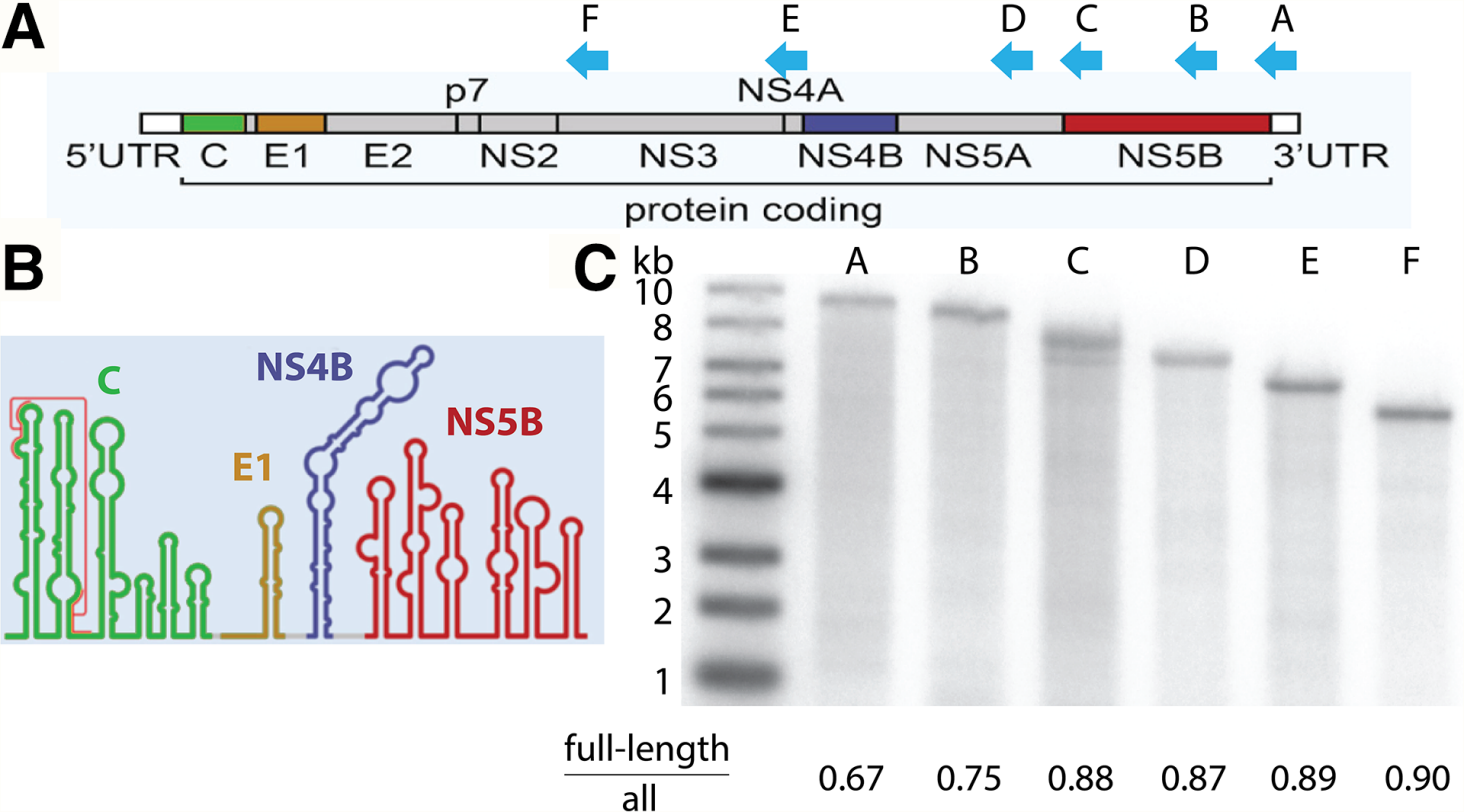
The RT reaction catalyzed by MarathonRT on a 9.6 kb viral genome. (*A*) Schematic diagram of HCV genome construction and (*B*) its secondary structure. Positions of primer binding sites (A [9461], B [8953], C [8051], D [7097], E [5912], F [4940]) are shown as blue arrows. (*C*) Representative denaturing alkaline agarose electrophoresis gel showing products of MarathonRT from multi-turnover RT reactions using full-length HCV genome as template. The ratio of signal intensity from full-length product divided by total products for each primer is indicated *under* each gel lane. The ladder is a double-stranded 1 kb DNA ladder (NEB), the mobility of which may be affected by incomplete denaturation in the gel.

In order to assess the relative processivity and reactivity of MarathonRT on a large, biologically relevant template, we examined cDNA synthesis from the genome of hepatitis C virus (HCV). Like many RNA viruses, the HCV genome is very long (∼9.6 kb, Fig. 2A) and it is among the most highly structured RNA genomes known (Davis et al. 2008), containing a multitude of stable architectural elements that regulate the pace of viral translation, replication, and packaging and other processes (Mauger et al. 2015; Pirakitikulr et al. 2016). This one RNA molecule, which contains long stem–loops, pseudoknots, and stable tertiary structures (Fig. 2B; Mauger et al. 2015; Pirakitikulr et al. 2016), presents all of the obstacles that can confound conventional retroviral RT enzymes (Harrison et al. 1998; Klasens et al. 1999). To evaluate relative cDNA synthesis efficiency by the MarathonRT, we extended reverse-transcription from six different sites in the genome (Fig. 2A,C), resulting in cDNA fragments that range from 4.9 to 9.5 kb in length (Fig. 2C). During this initial test, we used standard RT reaction conditions, in which the enzyme (500 nM) is in excess relative to the template (100 nM). However, unlike other RTs (which are typically used at high temperatures such as 50°C–70°C), we used the MarathonRT at its relatively low optimal temperature (42°C), despite the fact that lower temperatures stabilize RNA substructures.

Examination of the cDNA products reveals that all primers were extended efficiently and without prevalent stops, resulting in cDNAs ranging from 5000 to 9600 nt in length. The yield of full-length radiolabeled product was ∼90% for all fragments ≤8000 nt in length (Fig. 2C), which is an unprecedented efficiency of primer extension, particularly on such long templates. The primer extension yield provides a semi-quantitative metric of RT processivity, which begins to decline only for template lengths >8000 nt, although yields remain exceptionally high for these lengths. For example, the intact 9600-nt RNA template was copied with a total yield of 67%, which is more than sufficient for end-to-end sequencing of most viruses, pre-mRNA transcripts, or long noncoding RNAs.

### Comparative analysis of full-length primer extension capability

**FIGURE 3.**
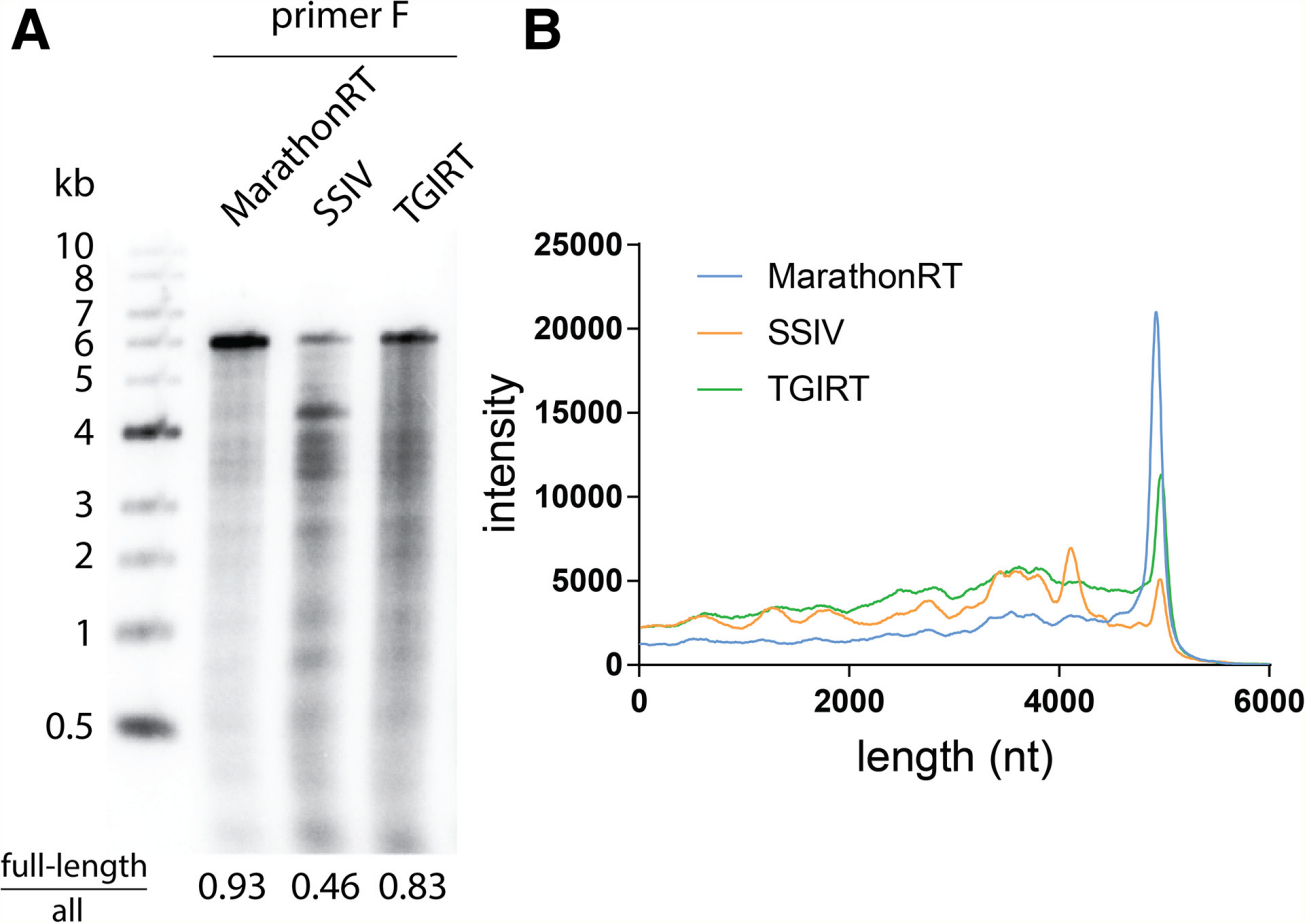
Comparison of RT products from MarathonRT, SSIV, and TGIRT using the HCV genome as template. (*A*) A representative denaturing alkaline agarose gel showing products from multiple-turnover RT reactions catalyzed by different polymerases. (SSIV) Superscript IV. The ratio of signal intensity from full-length product divided by total products for each polymerase are indicated *under* each gel lane, and were calculated as described in Materials and Methods. (*B*) Intensity profile for gel lanes in *A* that represent RT products produced by MarathonRT, SSIV, and TGIRT. RT reactions for SSIV and TGIRT were performed with optimal temperature (55°C for SSIV and 60°C for TGIRT) and standard buffer conditions according to the manufacturer's protocol.

In order to compare the capabilities of the MarathonRT with other high-performance RTs, we evaluated their relative ability to carry out full-length primer extension of kilobase RNA templates. Interestingly, there have been few head-to-head, quantitative comparisons of primer extension by RT variants that are commonly used for RT-PCR, SHAPE and DMS probing, and other applications. While TGIRT has been compared with SSII (Mohr et al. 2013) and retroelement RTs have been compared with AMV (avian myeoloblastosis virus) RT (Bibillo and Eickbush 2002), there have been no studies comparing the relative performance of RTs on long templates with direct analysis of first-strand cDNAs. To address this gap in understanding, we performed cDNA synthesis with a collection of high-performance RT enzymes [Superscript IV (SSIV), TGIRT, and MarathonRT] under their individual optimal reaction conditions using Primer F on the HCV genome, which results in a 4940-nt maximal-length product (Fig. 3). Consistent with our previous results, the MarathonRT produces 93% full-length product with few apparent stops. The SSIV produces 46% full-length product, although many strong stops are evident from a gel of extension products. Consistent with the fact that TGIRT also derives from a maturase, the template is copied reasonably well (83%), but the background level is high (Fig. 3). This could be due to reduced RT processivity, but it may be attributable to the high temperature required for the thermophilic TGIRT, which may induce template breakage. These results show that, using multiple-cycle conditions (RT enzyme excess), the MarathonRT performs exceptionally well compared with other high-performance RTs in copying a 5 kb viral RNA template.

### Comparative analysis of RT processivity under single-cycle reaction conditions

Under the multiple-cycle (enzyme excess) conditions that are typically used for primer extension by RT enzymes, partially extended fragments that result from RT dissociation can be rescued and further extended by reassociation with a different RT molecule. Therefore, the lengths of cDNA products under multiple-cycle conditions do not reflect the continuous extension activity of an individual RT molecule along RNA template (processivity). Formally, processivity is described as the probability that a polymerase will continue to copy the template rather than falling off. It describes the tendency of the polymerase to stay in the elongation mode and can be defined as the number of nucleotides incorporated during a single template-binding event (Bloom and Goodman 2001). Therefore, processivity must necessarily be measured under “single-cycle” conditions in which dissociated polymerase is prohibited from rebinding the template, thereby making it possible to determine the fraction of full-length cDNA products that are generated in a “single pass” by an RT enzyme.

**FIGURE 4.**
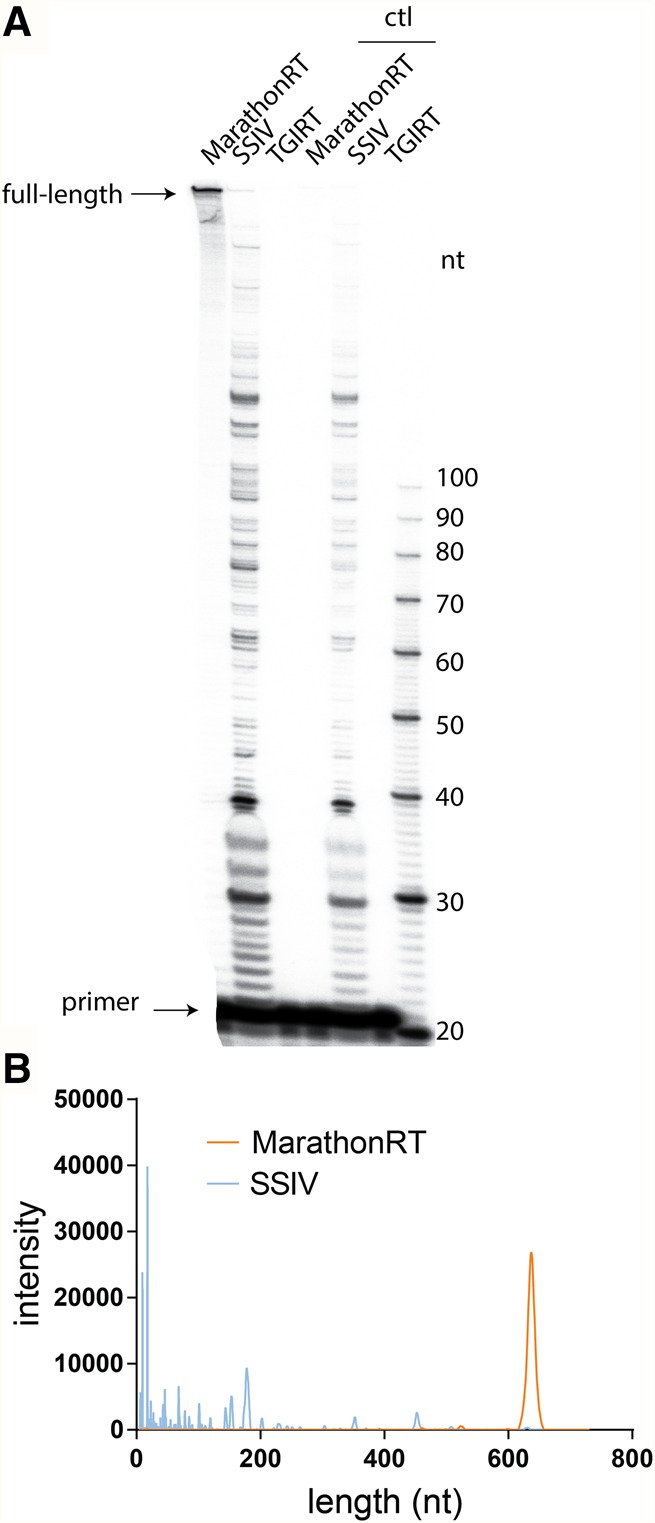
Single-cycle RT reaction on a noncoding RNA domain. In these experiments, a fragment from a long noncoding RNA (Domain 3 from lncRNA RepA [Liu et al. 2017]) was used as template, and any dissociated RT was trapped by the addition of a trap duplex (see Materials and Methods) upon initiating reaction. (*A*) Representative denaturing acrylamide gel of products. The “ctl” lanes indicate conditions in which RT is incubated with a vast excess of trap before being presented to the template. (*B*) Intensity profile for gel lanes that represent RT products produced by MarathonRT and SSIV.

To measure RT processivity under single-cycle conditions, an excess of trap (an RNA–DNA duplex resembling the primer binding site, see Materials and Methods) was added upon initiation of the extension reaction by the MarathonRT, SSIV, and TGIRT RT enzymes (Reddy et al. 1992). To sensitively monitor extension by diverse RT enzymes under these nonpermissive conditions, a template of moderate length and structural stability was chosen (D3 of lncRNA RepA, 643 nt) (Liu et al. 2017), and enzyme was added in slight excess over template concentration. The RT reactions for SSIV and TGIRT were performed under the optimal temperature and buffer condition as suggested by the manufacturer. After optimization of the trap concentration, reaction conditions and template, we identified a suitable set of single-cycle reaction conditions in which trap effectively prevents reassociation of the RT. This is demonstrated by the fact that cDNA products are not observed when trap is preincubated with the RT/template complex (rather than upon initiation of reaction with dNTPs, see Materials and Methods, Fig. 4A, control).

Under the single-cycle conditions, both MarathonRT and SSIV are capable of generating full-length cDNA product (Fig. 4A,B). Primer extension by TGIRT was not observed, perhaps because the high-temperature reaction conditions result in a dynamic initiation complex that is not stable enough to function under high trap concentrations (Fig. 4A,B). Alternatively, TGIRT may have a lower overall affinity or a lower active enzyme fraction that results in smaller fraction of active initiation complex, which would not be apparent when the enzyme is in excess, as in multiple-cycle conditions (Fig. 3). This could be a consequence of reduced enzyme solubility. Although SSIV can generate a small amount of full-length product under single-turnover conditions as indicated by the intensity profile (Fig. 4B), it primarily produces shorter fragments (Fig. 4A,B). In contrast, as observed under multiple-cycle conditions, MarathonRT copies the template end-to-end without any apparent stops and without generating any short fragments (Fig. 4). Thus, MarathonRT has a higher intrinsic processivity than either SSIV or TGIRT.

It is notable that SSIV still catalyzes primer extension reactions under the “control” reaction conditions, in which enzyme is preincubated with trap. In contrast, both the MarathonRT and TGIRT are fully trapped under these same conditions. These findings suggest that SSIV may have weak affinity for the trap. But more likely, given its efficiency under multiple-cycle conditions, SSIV appears to form a dynamic complex with template–cDNA hybrids, dissociating and reassociating rapidly from initiation sites and partial extension products, thereby ultimately driving polymerization to completion. Given these disparate behaviors by the RTs, the processivity derived for MarathonRT represents its actual processivity, whereas the processivity derived for SSIV can only be interpreted as an upper bound.

To quantify processivity from these experiments, we utilized the median of the cDNA length distribution to represent the “average” product length in a single RT reaction on a specific template, which is historically described as the macroscopic processivity (Wang et al. 2004). For the MarathonRT on the D3 template (for which the expected full-length cDNA product is 622 nt), the macroscopic processivity value is 616 ± 1 nt, which implies that, on this particular template, 99% of all enzymes that initiated reverse-transcription reached the end of the template (see Materials and Methods for calculation). From this value, one can estimate that the absolute processivity on a per nucleotide basis (the probability that the RT will extend one nucleotide rather than dissociate) is 99.998%, from which one can compute the mean template length at which 50% of the RT will dissociate before reaching the terminus (32,240 nt). While based on simplified assumptions, this value is very high, suggesting that the MarathonRT can be used to copy genomes as large as that of a coronavirus (∼30,000) in a single pass. In practice, processivity is likely to be lower, as it depends on structures and modifications of the template, reaction conditions, and other features that could cause the MarathonRT to dissociate prematurely.

Using this method, the measured macroscopic processivity of SSIV is only 19 ± 1 nt on the D3 template, suggesting that this enzyme is fundamentally nonprocessive and that dynamic reassociation of this RT is the force that drives the apparently efficient multiple-cycle reverse-transcription by this enzyme (Fig. 4A,B).

### Structural determinants of high RT processivity

**FIGURE 5.**
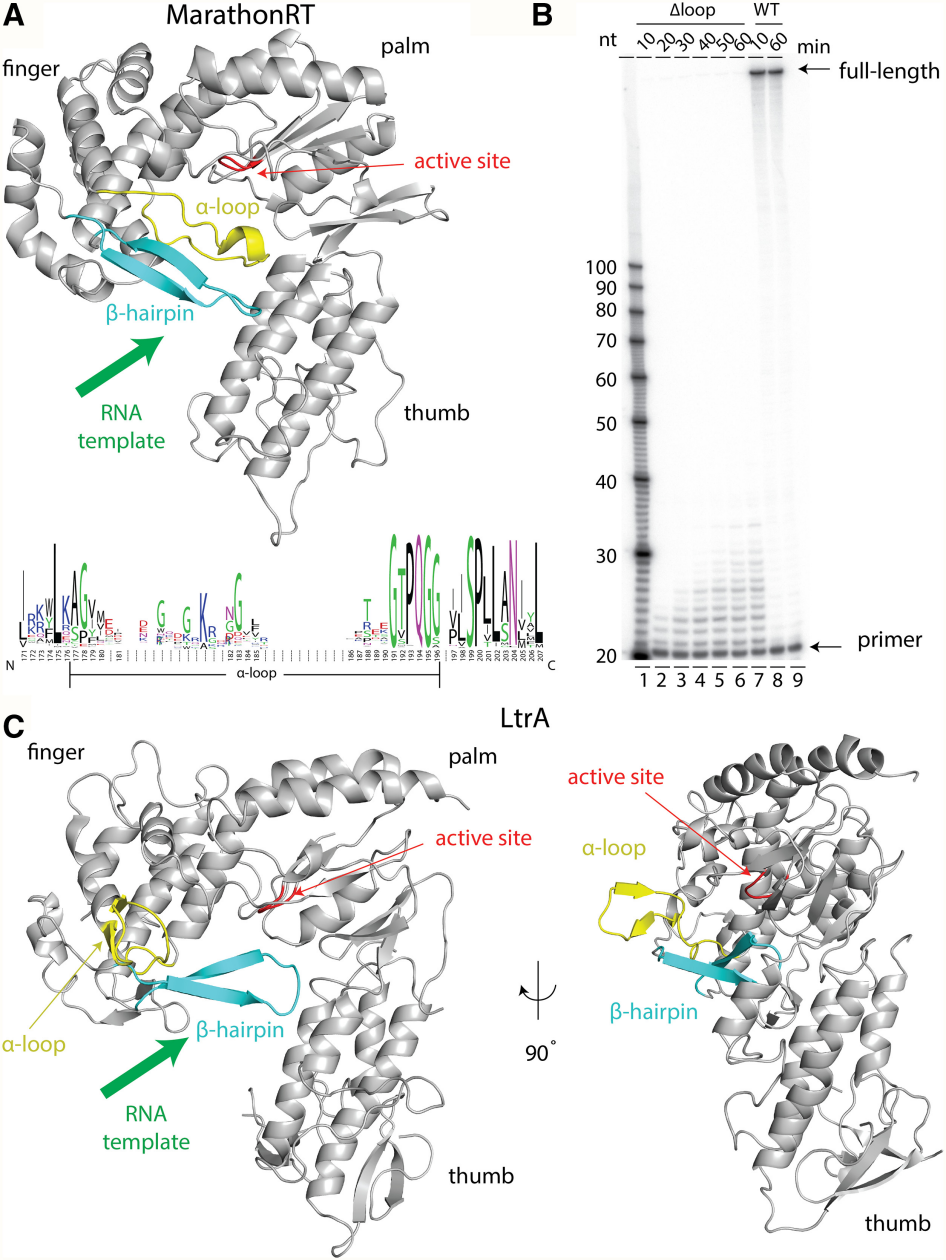
The α-loop is a processivity factor in group II intron maturases. (*A*) Three-dimensional model of the MarathonRT. The structure of the RT domain (finger and palm) was determined by X-ray crystallography (PDB ID: 5HHL), and the structure of thumb subdomain was created as a threaded model using I-TASSER (Yang et al. 2015) using the coordinates for LtrA (PDB ID: 5G2Y). A green arrow indicates the entry site for RNA template. The YADD motif that coordinates the active site Mg^2+^ ions is shown in red. *Below* is shown the sequence conservation for the α-loop and surrounding regions from all maturase sequences in the database (Candales et al. 2012), created using the web server WebLogo (Crooks et al. 2004). (*B*) Gel showing the RT products produced by WT and Δloop mutant of MarathonRT at different time points. The RNA template is Domain 3 from lncRNA RepA (Liu et al. 2017) (same template as in Fig. 4). (*C*) α-loop is in an open conformation in the cryo-EM structure of LtrA–LtrB intron complex (PDB ID: 5G2Y).

Given the unusually robust, processive behavior of MarathonRT, we set out to determine whether there might be a structural basis for its unique capabilities. Recent high-resolution structures of MarathonRT and other group II intron RTs (Qu et al. 2016; Zhao and Pyle 2016) have made it possible to conduct structure–function analyses on the specific motifs and substructures that are unique to this RT family and to determine whether they confer the extraordinarily processive RT activity that is observed (Figs. 1B, 5A).

From a kinetic standpoint, overall RT processivity is the result of competing forces that either drive the translocating polymerase forward, lead it to slide backward, or cause polymerase disassociation from the template (McClure and Chow 1980). As backward translocation is generally not observed (Yin and Steitz 2004; Bar-Nahum et al. 2005; Ó Maoiléidigh et al. 2011; Yu and Oster 2012), disassociation from the template is the major factor in reducing processivity of a polymerase. Therefore, structural features that promote high RT processivity are likely to be those that facilitate strong, productive interactions with RNA. For most common reverse transcriptases, the β-hairpin within the finger subdomain, together with residues in the thumb subdomain, enclose the RT active site, and prevent dissociation of the RNA template (Figs. 1B, 5A). For example, in HIV RT, extending the β-hairpin by 15 amino acids improved RT processivity (Kew et al. 1998). In addition, the thumb domain plays a key role in mediating polymerase processivity (Zhao and Pyle 2016).

Group II intron and non-LTR retrotransposon RTs have additional motifs that may enhance template binding and processivity. For example, a loop structure that is unique to this RT family (the α-loop) is located in the finger subdomain of the MarathonRT. This loop is proximal to the β-hairpin and it fully encloses the active site (Fig. 5A). Consistent with a role in processivity, deletion of the α-loop (the Δloop mutant) results in complete loss of long extension products, even under multi-turnover conditions (Fig. 5B). Behavior of the Δloop mutant is consistent with that of distributive polymerases, which frequently dissociate from the RNA template and then rebind. These results establish that the α-loop is a processivity motif in the MarathonRT, and based on sequence alignment (Fig. 5A; Zhao and Pyle 2017), it is present and likely to play a similar role in other group II intron maturases and closely related non-LTR RTs.

### Optimizing the MarathonRT

**FIGURE 6.**
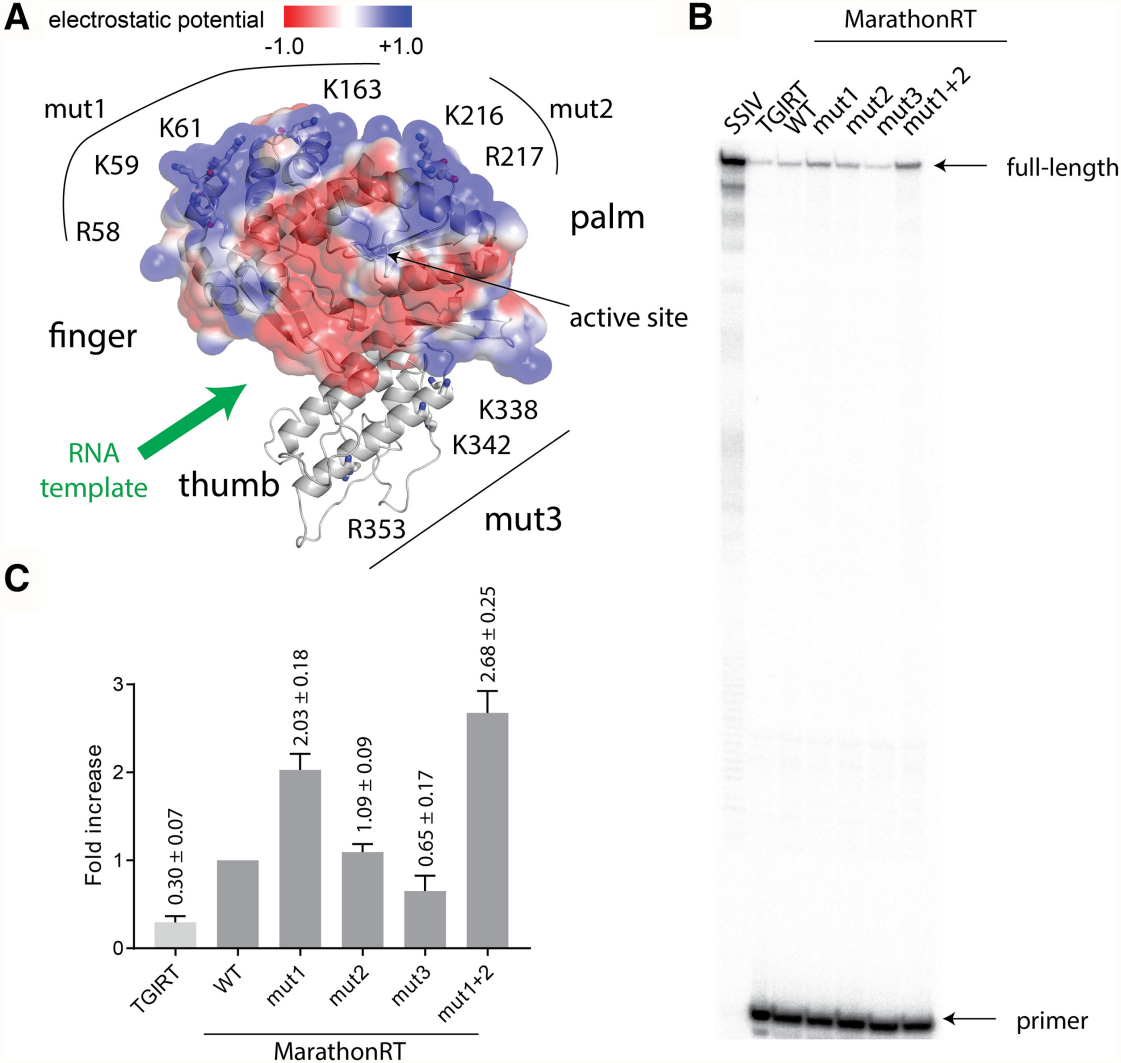
Positively charged RNA binding surface affects RT efficiency on lncRNA RepA D1. (*A*) Three-dimensional model (generated as described for Fig. 5A) showing the positively charged RNA binding surface (blue) on the RT domain of MarathonRT. The electrostatic surface potential of the RT domain was calculated using APBS (Baker et al. 2001) and PDB2PQR (Dolinsky et al. 2007) and is represented as a transparent surface. Residues that are mutated in mut1, mut2, and mut3 constructs were shown as sticks. (*B*) Gel showing the RT products produced by SSIV, TGIRT, and different constructs of MarathonRT using RepA D1 (Liu et al. 2017) as template under multi-turnover conditions. (*C*) Fold increase in the primer incorporation efficiency for various enzymes relative to WT MarathonRT. Primer incorporation efficiency is the ratio of all extension products relative to the total amount of primer in the reaction (equal to all extension products plus unincorporated primers).

Despite its unusual long-distance RT capabilities, it is important to keep in mind that the MarathonRT did not evolve to function exclusively as a polymerase. Rather, it evolved to stimulate group II intron splicing and retrotransposition, and it contains additional motifs that contribute exclusively to those functions (Fig. 6A; Matsuura et al. 1997; Wank et al. 1999; Qu et al. 2016; Zhao and Pyle 2016). Some of these motifs, such as the secondary RNA binding motif needed for tight interaction with the parent intron, are expected to detract from its ability to function as an RT, suggesting that the MarathonRT could be engineered to function more optimally as a tool enzyme. For example, we observe that, when a relatively unstructured RNA molecule (such as RepA domain 1, RepA D1) is used as the RNA template, MarathonRT utilizes only a small portion of primer (7.1% ± 1%). This is even more pronounced for TGIRT (2.1% ± 0.1%) (Fig. 6B). In contrast, SSIV extends 98.6% ± 1% of provided primer (Fig. 6B). Intriguingly, this primer utilization problem is not as severe in RT reactions involving more structured templates, such as RepA D3 (Supplemental Fig. 1). One explanation for this phenomenon is that certain templates (and perhaps the primer) may bind to the highly basic secondary RNA binding site that is located on the surface of the RT (Fig. 6A). This secondary site may trap the RNA and the primer in an unproductive binding mode, essentially removing it from the pool of active complexes.

To test this hypothesis experimentally, we created mutants that reduce the positive charge on the secondary RNA binding site of MarathonRT and we measured the primer incorporation efficiency using RepA D1 as template. The crystal structure of the *E.r.* RT domain (Zhao and Pyle 2016), and the cryo-EM structure of a related group II intron-maturase complex from *L.l.* (Qu et al. 2016), revealed a highly positively charged region that is located on the protein surface opposite the RT active site (Fig. 6A). Given that this region is unlikely to play a role in reverse-transcription, we speculated that it could be modified to reduce nonspecific binding. For example, we created a mutant that modifies amino acids on one lobe of the secondary binding surface, including R58A, K59A, K61A, and K163A (mut1). This mutation is expected to eliminate the maturase region that normally binds intron RNA motif D4A (Fig. 6A). Another set of mutations (mut2) on the second lobe includes K216A and R217A, which are expected to interact with intron D1. We also combined these into a single mutant that contains all six basic amino acid changes (mut1 + mut2). Finally, we designed a set of mutations on the maturase thumb domain (mut3, including K338A, K342A, and R353A) (Fig. 6A) that are predicted to interact with 5′exon for facilitating group II intron splicing. In keeping with a reduction in nonspecific primer binding, the mut1 construct displays a 2.03(±0.2)-fold increase in primer incorporation efficiency relative to the wild-type maturase, the mut2 construct has almost no change (1.09[±0.09]-fold increase), whereas mut1 + mut2 construct has a 2.67(±0.25)-fold increase in productive primer binding relative to the wild-type enzyme (Fig. 6B,C). This gradual increase in primer incorporation efficiency by decreasing the positive charge on the intron binding surface suggests that template and/or maturase depletion is likely to play a role in the primer incorporation problem. Additionally, this nonadditive improvement of mut1 + mut2 construct compared to mut1 and mut2 alone suggests that the nonproductive template binding is synergistic. However, even after incorporating six alanine mutations on the positively charged surface, the mut1 + mut2 construct is still only able to utilize 19% ± 3% of RepA D1 template. This suggests that additional structural features obstruct productive primer binding within the MarathonRT, and that more structure–function investigation is needed. Finally, mut3 has a 0.65(±0.17)-fold decrease compared to the wild type (Fig. 6B,C), suggesting that the positively charged residues that interact with the 5′ exon during group II intron splicing, also play a role in recruiting RNA template during RT reaction.

### Fidelity of the MarathonRT

We anticipate that MarathonRT will be of particular utility during next-generation sequencing (NGS) library preparation from RNA. As such, it is important that it maintains high fidelity during reverse transcription. In order to determine the misincorporation frequency (often referred to as the error rate) of the MarathonRT, and compare it directly with SSIV and TGIRT, it was necessary to develop a suitable experimental approach that was accurate and free of PCR bias. The calculated percentage of misincorporation, which is frequently referred to as the “error rate” in other studies, has historically been estimated in various ways. For example, in the pre-NGS era, the *lacZ* mutation selection assay was the most widely used method (Kunkel 1985), and it has been used in more recent studies as well (Mohr et al. 2013). This approach underestimates the misincorporation frequency since the genetic code is degenerate/redundant, resulting in silent mutations that retain a functional *lacZ* protein. More recently, high-throughput sequencing has been used to monitor fidelity by directly counting the mutational frequencies in the sequencing reads (Mohr et al. 2013). However, this method is sensitive to PCR bias, as it cannot discriminate RT error from subsequent PCR amplification or base-call errors derived from the sequencing platform (Lee et al. 2016). Therefore, traditional high-throughput sequencing is not ideal for accurate estimation of RT misincorporation frequency.

**FIGURE 7.**
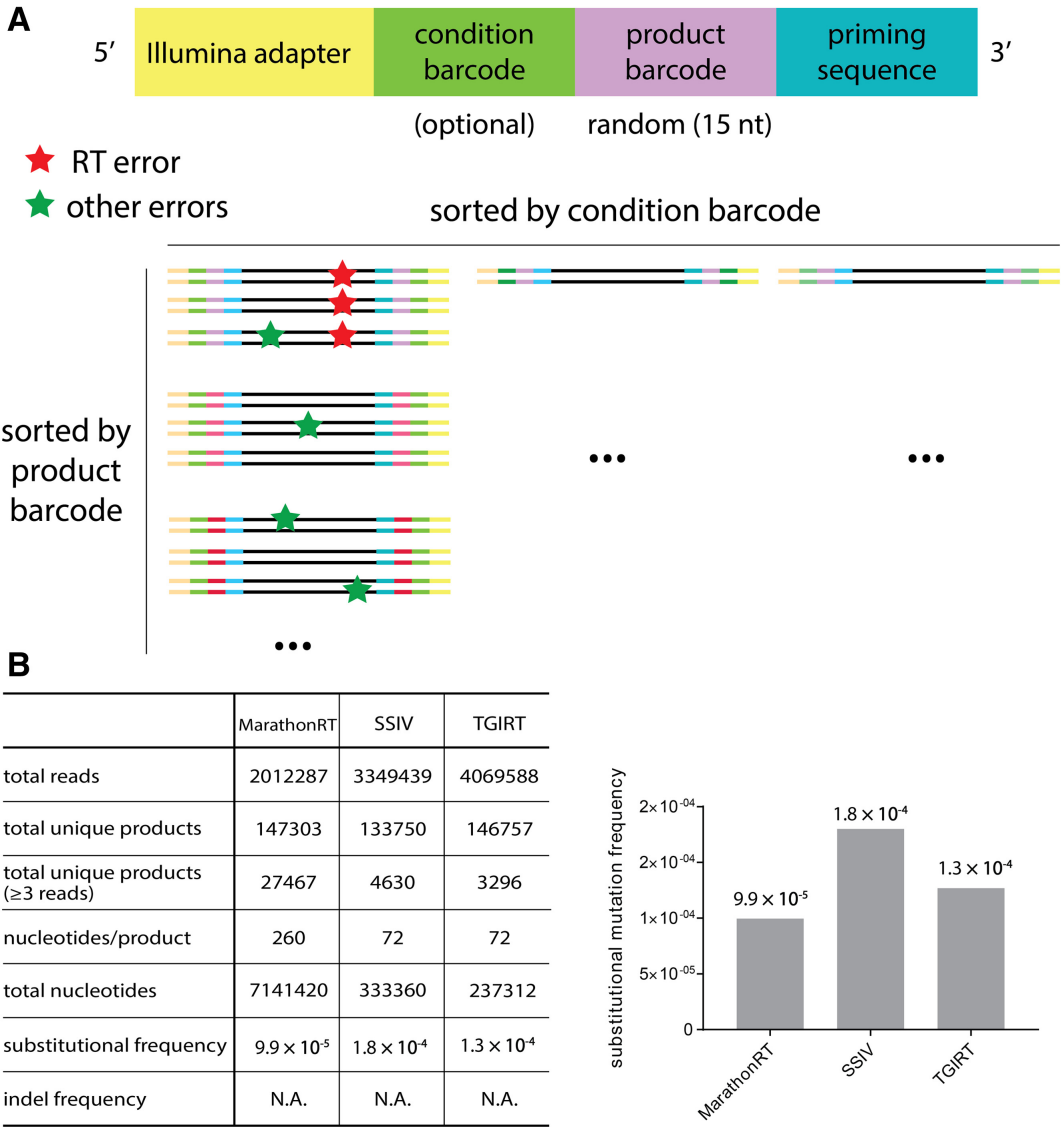
Error rate of various reverse transcriptases including MarathonRT, SSIV, and TGIRT. (*A*) Single-molecule sequencing method. The schematic diagram of primers used for RT and second-strand synthesis is shown on the *top*. The principle underlying single-molecule sequencing is shown on the *bottom*. Only errors that are consistent in all sequencing reads that share the same product barcode are considered as RT errors (red stars). Errors that are inconsistent among reads sharing the same product barcode (green stars) will have originated from the PCR amplification or sequencing platform. (*B*) Error rate determination for different reverse transcriptases. The table summarizing the sequencing data is shown at *left*. In this table, nucleotides/product (row 4) is the number of nucleotides in each RT product that are used for final analysis, after the low quality bases at the ends were trimmed. Total nucleotides (row 5) is the total number of nucleotides involved in the analysis. The total number of reads (row 2) is the raw number of sequencing reads in either forward (R1) or reverse (R2) direction for each polymerase. The unique product (row 3) is a set of sequencing reads that share the same UMI (unique molecular identifier), and only unique products that have no less than three reads were included in the analysis (row 4). Nucleotide/product (row 5) shows the number of nucleotides that are incorporated by each polymerase after trimming the primer region and low-quality nucleotides at the end. Total nucleotides (row 6) is calculated by multiplying nucleotide/product (row 5) with the number of unique products (row 4), which is the total number of nucleotides analyzed. Substitution frequency (row 7) was calculated by dividing the number of total nucleotides (row 6) by the number of mutated nucleotides. Indel (insertion–deletion) frequency (row 8) was calculated by dividing the number of unique products by the number of indel events. N.A. suggests that current sequencing depth is not able to detect indels (insertion–deletion). The bar plot showing the substitutional frequency for MarathonRT, SSIV, and TGIRT is shown on the *right*.

To mitigate these issues, we adapted a single-molecule high-throughput sequencing method that had previously been used to study the fidelity of DNA polymerases (Lee et al. 2016), using it to determine the misincorporation frequency of the MarathonRT, SSIV, and TGIRT RT enzymes (Fig. 7). To faithfully monitor errors incorporated only by the RT, we incorporated a random 15-nt-long product barcode, or unique molecular identifier (UMI), at both ends of each RT product (Fig. 7A; Lee et al. 2016). Sequencing reads were then sorted via their product barcodes, and only mutations within all reads sharing the same barcode (PCR duplicates resulting from a single RT product) were considered as RT errors (Fig. 7A; Lee et al. 2016). This powerful barcoding method can distinguish errors from various sources, and it is free of PCR bias. By adapting this experimental approach, we measured the misincorporation frequencies for MarathonRT, in parallel with commercial SSIV and TGIRT. The substitutional mutational frequency determined from these unique RT products are 9.9 × 10^−5^ for MarathonRT, 1.8 × 10^−4^ for SSIV and 1.3 × 10^−4^ for TGIRT (Fig. 7B). This result suggests that the MarathonRT is as accurate as other high-fidelity reverse transcriptases such as SSIV and TGIRT.

Notably, the error rate we determined for TGIRT is about 10 times higher than that previously reported (Mohr et al. 2013), which is probably attributable to methodological differences between the two studies. In the previous study, which was conducted at the transcriptome-wide level, the authors computed data only from overlapping regions of forward and reverse reads in a pair-end sequencing experiment, discarding mutations that are common to both TGIRT and SSIV (Mohr et al. 2013). Given the active-site similarities among both classes of enzyme, this likely results in underestimation of misincorporation frequency for both enzymes.

## DISCUSSION

Here we report an unusually processive metazoan RT with features that will enable it to become a valuable new tool for sequencing and biotechnology. It is representative of a large family of RTs that show great promise but which have not been subjected to extensive mechanistic analysis. Identification and characterization of this RT is the outgrowth of structural and biochemical studies that have enabled us to ascertain the molecular basis for its unusual processivity and to optimize properties of this enzyme family.

In this study, we have shown that the reverse transcriptase within the MarathonRT enzyme displays exceptionally high processivity, as it can synthesize an entire HCV viral genome (∼9.5 kb cDNA) with few detectable stops. Such extreme RT processivity opens the door to new RNA sequencing methods and new approaches for studying large RNA structures. For example, it may now be possible to sequence entire transcriptomes simply by priming the RT reaction with oligo(dT), thereby reducing the non-uniformity of read coverage and allowing more accurate quantification of mRNA expression levels (Archer et al. 2016). Because it can copy long RNA molecules end-to-end, the MarathonRT may be useful for characterizing heterogenous RNA populations when combined with third-generation end-to-end sequencing platforms (Chaisson et al. 2015; Jain et al. 2016). This would allow investigators to identify and quantify alternative splicing isoforms (Bolisetty et al. 2015), correlated mutations in viral quasi-species (Wu et al. 2014; Routh et al. 2015), variation in RNA-editing among different transcripts (Levanon et al. 2004; Meyer et al. 2012), and variations in secondary structures (Siegfried et al. 2014; Zubradt et al. 2017).

The MarathonRT has additional ramifications for biotechnology methods development. Direct RNA sequencing with processive enzymes would reduce the bias and artefacts that are associated with the RT-PCR step in current DNA-based sequencing protocols, thereby providing a more accurate reflection of gene expression pathways (Ozsolak et al. 2009; Vilfan et al. 2013). Because the MarathonRT is very stable in solution (Supplemental Fig. 2; Zhao and Pyle 2016), it would be readily incorporated into the SMRT sequencing platform developed by Pacific Biosciences (Eid et al. 2009; Korlach et al. 2010). Such long-read direct RNA sequencing techniques would be particularly useful for single-cell RNA-seq experiments in which the RNA species are heterogeneous.

Building on crystallographic studies of the MarathonRT (Zhao and Pyle 2016), we identified certain structural features that promote the high processivity of this enzyme family. Specifically, the MarathonRT contains a loop motif (the α-loop) that encloses the RT active site and prevents RNA template dissociation (Fig. 5C; Zhao and Pyle 2016, 2017). This loop is highly conserved among all group II intron maturases (Fig. 4A), suggesting that it plays a universal role in ensuring RT processivity. Importantly, the amino acid sequence of the α-loop is poorly conserved (Fig. 5A), suggesting that it functions as a steric block. The presence of the α-loop in non-LTR retrotransposon RTs such as L1 indicates that α-loop-mediated RT processivity is broadly conserved and likely to also play a role in other non-LTR RTs.

Conformational dynamics of the α-loop may help to regulate RT activity. The crystal structure of the finger and palm regions of MarathonRT was obtained in the absence of RNA template, and in that context, the α-loop forms a short α-helix at its tip and it adopts a closed conformation that obstructs the RNA template entry pathway (Fig. 5A,C; Zhao and Pyle 2016). In contrast, in the cryo-EM structure of a related group II intron maturase (LtrA) (Qu et al. 2016), the same region of this loop forms a β-hairpin that is stabilized in an open conformation through interactions with intron domain 4 (D4) (Fig. 5C). This observation suggests that interactions with group II intron RNA may regulate the RT activity of maturase proteins. In solution, the α-loop is likely to be flexible, which would accommodate the association of RNA template. Engineering the length and sequence of the α-loop may therefore facilitate the design of even more processive reverse transcriptases.

While investigating the mechanistic features of the MarathonRT, it was also important to benchmark the enzyme by comparing it with other high-performance RT enzymes. In general, our results suggest that the MarathonRT surpasses the performance of the related TGIRT enzyme, potentially because the MarathonRT was obtained from an informatic screen of biophysically stable enzymes. Although TGIRT has superior thermal stability, its solubility is low and it remains in solution only when fused C-terminal to a MBP (maltose binding protein) tag (Fig. 1A; Mohr et al. 2013). Perhaps because of these issues, the primer utilization efficiency of TGIRT is low (∼30% of MarathonRT WT and ∼11% of MarathonRT mut1 + mut2 construct, Fig. 6B,C). In addition, extension products are not observed for TGIRT under the single-turnover conditions investigated here (Fig. 4A), which may prevent its application in direct RNA sequencing.

The wild-type MarathonRT has an important shortcoming that we identified upon comparison with SSIV. Despite its low processivity, SSIV displays a remarkably high primer utilization efficiency relative to the MarathonRT (Fig. 6B). These data indicate that different RT enzymes have varying strengths and weaknesses, and that one should select a reverse transcriptase that is ideally suited for a given task. For example, if primer utilization efficiency is the primary goal, as in RNA diagnostics and conventional RT-qPCR, SSIV is an excellent choice for an RT enzyme. However, if faithful end-to-end RNA sequencing is the priority, or if one is working with a highly structured RNA template, the MarathonRT is likely to be the best candidate available. If one needs to conduct the RT reaction at high temperatures (>60°C), TGIRT will be the ideal choice.

In this paper, we have focused on the inherent attributes of the wild-type MarathonRT, but like commercial preparations of SSIV and TGIRT, it should be possible to optimize this enzyme and improve its properties. For example, using insights from the crystal structures of the MarathonRT, we designed variants with altered primer utilization efficiency. Neutralization of positive charges on the surface of the protein resulted in modest improvements in RT efficiency (approximately threefold increase in primer incorporation efficiency). However, the efficiency of this engineered MarathonRT is still only 20% of SSIV on the RNA template tested, so additional alterations to the design will be needed for further improvements in RT efficiency. It remains possible, however, that low enzyme turnover rate (which contributes positively to processivity) plays a negative role in RT efficiency, suggesting that new strategies will be required for building an RT enzyme that is both hyper-processive and efficient at primer utilization.

The mutational frequency of the MarathonRT is comparable to that of other high-fidelity RTs such as TGIRT and SSIV (∼1 × 10^−4^). Although this substitutional frequency is an order of magnitude larger than high-fidelity proofreading DNA polymerases such as *Pfu* and Q5, it is comparable to the error rate of Klenow fragment, which also lacks a proofreading exonuclease domain, and it is comparable to that of *Taq* polymerase, which has proofreading activity (Lee et al. 2016). Therefore, the error rate of MarathonRT is about the best that a polymerase can achieve without a proofreading exonuclease domain. However, we measured the error rate using a single RNA template, so it remains possible that the error rates on other RNA templates are slightly different. Nevertheless, by focusing on a single RNA template and using a single-molecule barcoding strategy, we obtained highly accurate data that are free of PCR bias. In the future, similar experiments using a set of “representative” RNA templates would provide a more complete understanding of the confidence intervals in the error rate.

In summary, we have demonstrated that the MarathonRT displays exceptional levels of processivity under both single and multiple-turnover conditions, and that it has an error rate that is typical of high performance RT enzymes. We have identified structural features that contribute to the enhanced capabilities of the MarathonRT and we have manipulated these to modulate behavior of the enzyme. Finally, we have benchmarked the MarathonRT and compared its attributes to behavior of other common RT enzymes, showing that each of these enzymes has strengths and weaknesses that can impact their application in common technical procedures. The addition of a soluble, stable, and hyper-processive metazoan RT to the collection of available enzymes will greatly advance RNA biotechnology and research, which is particularly important at this time of intense interest in long RNA molecules and their role in biology.

## MATERIALS AND METHODS

### Construct descriptions, protein expression, and purification

The protein sequence for wild-type (WT) *E.r.* maturase (from group IIC intron Eu.re.I2, henceforth called MarathonRT) was obtained from the group II intron database (Candales et al. 2012), where the sequence corresponds to regions 124,807–126,667 in GenBank accession entry FP929043.1. The codon-optimized cDNA was synthesized by Invitrogen (Thermo Fisher). All mutant constructs were generated using a Q5 Site-Directed Mutagenesis Kit (NEB). Construct mut1 is a quadruple mutant consisting of R58A, K59A, K61A, and K163A; construct mut2 is a double mutant consisting of K216A and R217A. Construct mut1 + mut2 contains all six of the previously mentioned point mutations (i.e., it is a combination of mut1 and mut2). Construct mut3 is a triple mutant that consists of K338A, K342A, and R353A. In the Δloop mutant, residues 182–192 have been replaced with two glycines.

Protein expression and purification were performed as described previously (Zhao and Pyle 2016) with the following modifications: After SUMO tag cleavage, protein was directly loaded onto a 5 mL Hitrap SP column (GE Healthcare) equilibrated with a K-HEPES buffer containing 300 mM KCl at pH 7.5 (low salt buffer). Hitrap SP provided improved resolution for some of the maturase variants relative to the Hitrap Heparin column that has been used in our earlier work (Zhao and Pyle 2016). For WT, mut1, mut2, and mut3 MarathonRT constructs, bound proteins were initially eluted with a K-HEPES buffer containing 2 M KCl at pH 7.5 (high salt buffer). The peak fraction (in 5 mL) was diluted to 70 mL with low salt buffer, and then loaded onto the Hitrap SP column equilibrated with a mixture of 72% low salt buffer and 8% high salt buffer. The bound protein was eluted with a linear salt gradient that reaches 50% high salt buffer after 50 mL elution (starting from 8% high salt). For the mut1 + mut2 construct, the supernatant was loaded on the column after clarifying the SUMO tag-cleavage reaction, and then the protein was eluted with a linear salt gradient that reaches 50% high salt buffer after 50 mL elution (starting from 0% high salt). For all constructs, after Hitrap SP purification, the proteins were passed over a Superdex S200 Increase column (10/300 GL, GE Healthcare), and the peak fraction was pooled, concentrated to 2–20 mg/mL, and flash-frozen in liquid nitrogen.

### Multiple-cycle RT assays

In these experiments, RepA D1 (residues 1–419), RepA D3 (residues 998–1630) (Liu et al. 2017), or the intact HCV genome (strain Jc1) (Pirakitikulr et al. 2016) were used as RNA templates, as indicated. The primer for RepA D1 annealed to position 387, the primer for RepA D3 annealed to position 1630, and primers for the HCV genome annealed to positions 4940 (F), 5912 (E), 7097 (D), 8051 (C), 8953 (B), and 9461 (A) (Supplemental Table 1). Each RT primer was 5′ end labeled with ^32^P by T4 PNK, and the labeled primer was purified on a 20% polyacrylamide gel. Final RNA template concentration was 100 nM and the final RT enzyme concentration was 500 nM. The 1× RT reaction for MarathonRT contained 50 mM K-HEPES (pH 8.5), 100 mM KCl, 2 mM MgCl_2_, and 10 mM DTT. The RT reactions for SSIV and TGIRT were set up according to the manufacturer's protocol. Reactions were incubated at 42°C for MarathonRT, 55°C for SSIV, and 60°C for TGIRT. Reactions were allowed to proceed for 10 min in the case of RepA D1 and D3 templates, and 1 h in the case of the HCV genome. The RT reactions were stopped by heating them at 95°C for 1 min, after which the RT enzymes were then digested by protease K, and RNA templates were hydrolyzed by 300 mM NaOH before analyzing cDNA products.

First-strand cDNA products synthesized from the RepA D1 and D3 templates were resolved on a 10% polyacrylamide sequencing gel along with a ssDNA ladder (Simplex). The first-strand cDNA products synthesized from the HCV genome were resolved on a 0.8% (w/v) alkaline agarose gel (SeakKem LE) according to published protocol (Sambrook and Russell 2006). Gels were run in 1× alkaline gel running buffer at room temperature for 5 h at 2 V/cm. They were then transferred onto a Hybond-N^+^ nylon membrane (GE Healthcare), which was placed on top of two layers of Whatman paper and then covered with Saran wrap. To avoid cracking, the gel was first dried at 80°C for 1 h under vacuum, and then it was allowed to slowly cool to room temperature under vacuum for another 1 h. The size ladder was a 1 kb double-stranded (ds) DNA ladder (NEB) that was denatured under alkaline gel-electrophoresis conditions.

### Single-cycle processivity assay

The RepA D3 RNA (residues 998–1630) (Liu et al. 2017) was the template for single-cycle processivity assays, and the RT primer was annealed to the extreme 3′ end (Supplemental Table 1). RT primers were 5′ end labeled with ^32^P by T4 PNK, and then purified on a 20% polyacrylamide gel. Before use, RNA templates were first diluted to 100 nM (10× stock) in an RNA storage buffer containing 10 mM K-MES (pH 6.0) and 1 mM EDTA. The RNA template was then mixed with 100 nM (10× stock) primer at 1:1 volume ratio, and the mixture was heated to 95°C for 1 min, and then snap cooled on ice for 10 min. The annealed primer–template was incubated with 400 nM RT enzymes (10× stock) in reaction buffer as follows: For MarathonRT, 2 µL template–primer mixture was combined with 2 µL H_2_O and 1 µL 10× RT reaction buffer (500 mM K-HEPES pH 8.5, 1 M KCl, 20 mM MgCl_2_, 100 mM DTT). For SSIV and TGIRT, 2 µL of the template–primer mixture was combined with 1 µL DTT (100 mM), and 2 µL 5× RT reaction buffer (commercial). In each case, incubation was performed at room temperature for 10 min prior to initiating reaction by the addition of dNTPs. For single-cycle reactions, a trap (RepA D1 annealed to a primer at position 387 [Supplemental Table 1]) was added to 10 µM final concentration simultaneously with KCl to 400 mM and dNTPs to 0.5 mM. The RT reaction was performed for 5 min at 42°C for MarathonRT, 55°C for SSIV, and 60°C for TGIRT. The RT reaction was stopped by heating the samples at 95°C for 1 min to denature the enzyme, and the cDNA products were treated with proteinase K and 300 mM NaOH as described above to remove proteins and RNA template. First-strand cDNA products were resolved on a 10% polyacrylamide sequencing gel. For the control group, a similar procedure was followed except that trap (10 mM RepA D1 annealed to a primer at position 387 [Supplemental Table 1]) was included in the preincubation step for annealed template–primer and RT enzymes.

The intensity profile for each gel lane was extracted using ImageQuant TL software (GE Healthcare). Background was substracted by using a rolling-ball algorithm with 100 µm radius (within ImageQuant TL) to estimate the amount of background at each position. The corresponding pixel position for the median of intensity profile on each gel lane was calculated by a homemade script (available upon request). Pixel positions were converted to DNA length by interpolating the linear regression of the logarithm of lengths in single-stranded (ss) DNA ladder (Simplex) against pixel position. A rolling ball radius no smaller than 100 µm was deemed reasonable because the pixel size of the scanned gel is 100 µm. All plots were produced using Prism software (GraphPad, version 7.01), from three independent experiments. For extrapolating the template length which gives 50% full-length cDNA product, we first estimated the probability of polymerase dissociation at each nucleotide by assuming that dissociation events at each nucleotide are independent of each other, and that the probability of polymerase dissociation at each nucleotide position is the same. Therefore, when the full-length cDNA is 622 nt and the median of cDNA products is 616 nt, it suggests that 0.965% of primer extension events did not go to completion because of polymerase dissociation. When we assume that this total dissociation event is evenly distributed on the 622-nt full-length cDNA, this gives a 1.55 × 10^−5^ probability of disassociation at each nucleotide (equivalent to 99.998% processivity at per nucleotide basis). In this case, a template of 32,240 nt is required in order to have 50% disassociation event. However, given the limitations on resolution of the gel at position 616 nt, it is possible that the calculated processivity is slightly overestimated.

### Determination of misincorporation frequency

These experiments utilized the RepA D3 RNA (residues 998–1630) as template, with an RT primer that anneals to position 1398. Upstream of the annealing site, the RT primer also contains 15 nucleotides (nt) of random sequence (a unique molecular identifier, or UMI) that is followed by a 4-nt condition barcode and a region complementary to the Illumina universal primer located at the very 5′ terminus of the primer oligonucleotide (Fig. 6A; Supplemental Table 2). The primer used for second-strand synthesis has a similar configuration, as it contains a region complementary to Illumina Index primer at the very 5′ end, followed by a 3-nt condition barcode and region that is complementary to the extreme 3′ terminus of the first-strand cDNA (Fig. 6A; Supplemental Table 2). In principle, the condition barcode was designed to sort different reaction conditions, but in this study, we simply used the same condition barcode for all enzymes, and different enzymes were barcoded by Illumina indexes.

RT reactions were conducted in 20 µL final volumes using 0.2 pmol RNA template (1.2 × 10^11^ molecules) annealed to 0.2 pmol RT primer, which is much less than the number of molecules that can be encoded by combined UMIs from both primers (15 nt each, 4^30^ = 1.15 × 10^18^). The RT reactions were performed under conditions similar to those described in the multi-turnover RT assay, except that the reaction time was 1 h. Reactions were stopped by heating to 95°C for 3 min, and then they were cooled slowly to allow efficient reannealing of first-strand cDNA to the RNA template. The RNA template was then digested by adding 1 µL RNase H (NEB) to the reaction mixture, which was then incubated at 37°C for 30 min. The reaction mixture was then combined with 0.2 pmol second-strand synthesis primer (Supplemental Table 2), and the second-strand cDNA was then synthesized by high-fidelity Q5 (NEB) in a 50 µL final reaction volume in a thermal cycler set for a single cycle (denature at 98°C for 20 sec, anneal at 50°C for 30 sec, and extend at 72°C for 20 min). The double-stranded (ds) cDNA products were then purified on 90 µL AMPure XP beads (Beckman) according to manufacturer's protocol. The ds-cDNAs were then eluted in 30 µL H_2_O, and their concentration was estimated by qPCR using a LightCycler SYBR Green I Master Kit (Roche) and plasmid DNA as standard. The ds-cDNAs were then adjusted to the same concentration (5 × 10^−15^ M) in different groups, and 1 µL of each dsDNA (3 × 10^9^ molecules) was amplified with PCR amplification primers (Supplemental Table 2) for 10 cycles in 25 µL PCR reactions. The PCR products were then purified with 45 µL AMPure XP beads (Beckman) and eluted in 15 µL H_2_O. After this cleaning step, 1 µL of each PCR product was further amplified in PCR reactions (25 µL total volume) for 13 more PCR cycles using the Illumina universal primer and Illumina index primers (NEBNext). For all PCR amplification steps, the PCR program involved denaturing at 98°C for 5 min, amplifying using the three-step protocol with desired cycle numbers (denature at 98°C for 20 sec, anneal at 64°C for 30 sec, and extend at 72°C for 30 sec), and finally extending at 72°C for 5 min. The specificity of PCR reactions was confirmed using an agarose gel stained with PicoGreen (Invitrogen). Finally, the PCR-amplified products were pooled and samples for MarathonRT were sequenced on an Illumina Miseq sequencer in paired-end mode for 250 cycles (PE250) with 20% PhiX spike-in, whereas samples for SSIV and TGIRT were sequenced on an Illumina Hiseq sequencer in paired-end mode for 75 cycles (PE75) as 1% spike-in at YCGA. The sequencing data were processed using published scripts (Lee et al. 2016). In brief, the primer binding region and low-quality residues at both ends (30 residues in R1 and 180 residues in R2) were first trimmed, and sequencing reads having residues with Q-score lower than 20 were discarded. The sequencing reads were then sorted based on the UMIs at both the 5′ end and 3′ end, and reads that share the same UMIs were counted as a unique product. Reads were aligned to the reference sequence using MUSCLE (Edgar 2004a,b), and errors were recorded only when the same substitutional mutation or insertion–deletions (indels) were observed in all reads that belong to the same unique product group. Only RT products with UMIs that appear no less than three times were used in estimating substitutional frequency.

In our analysis (summarized in Fig. 7B), there were 2,012,287, 3,349,439, and 4,069,588 reads sequenced in total for MarathonRT, SSIV, and TGIRT, respectively. Among these reads, there were 147,303 (MarathonRT), 133,750 (SSIV), and 146,757 (TGIRT) unique product barcodes, meaning that this number of individual RT reactions were analyzed. Among these unique RT products, 27,467 (MarathonRT), 4630 (SSIV), and 3296 (TGIRT) products have no less than three reads sequenced (at least three reads with the same product barcode/UMI), and are therefore included in the downstream analysis. The low-quality bases at the ends were then trimmed, resulting in only 260 nucleotides for MarathonRT and 72 nucleotides for SSIV and TGIRT. Then, low quality sequences were further eliminated by discarding all reads that have one or more nucleotides with a *Q*-score lower than 20. After this extensive quality filtering, there were 7,141,420 (MarathonRT), 333,360 (SSIV), and 237,312 (TGIRT) total nucleotides included in the final alignment and calculation of substitutional frequency, which is 9.9 × 10^−5^ for MarathonRT, 1.8 × 10^−4^ for SSIV and 1.3 × 10^−4^ for TGIRT. These numbers suggest that the total number of nucleotides sequenced in our experiment is sufficient to estimate the substitutional frequency. In our data, we did not observe insertion-deletion events (indel) for any enzyme.

## SUPPLEMENTAL MATERIAL

Supplemental material is available for this article.

## Supplementary Material

Supplemental Material
