# Supplementary material for: An ultraprocessive, accurate reverse transcriptase encoded by a metazoan group II intron
Source: RNA. 2018 Feb;24(2):183–95. doi: 10.1261/rna.063479.117 (PMC5769746; doi:10.1261/rna.063479.117)
Supplement: Supplemental Material [file supp_24_2_183__index.html]

An ultraprocessive, accurate reverse transcriptase encoded by a metazoan group II intron — Supplemental Material 

# An ultraprocessive, accurate reverse transcriptase encoded by a metazoan group II intron

## Supplemental Material

- Supplemental\_Fig\_S1.pdf
- Supplemental\_Fig\_S2.pdf
- Supplemental\_Table\_S1.pdf
- Supplemental\_Table\_S2.pdf
