# Supplementary figures and images for: An ultraprocessive, accurate reverse transcriptase encoded by a metazoan group II intron
Source: RNA. 2018 Feb;24(2):183–95. doi: 10.1261/rna.063479.117 (PMC5769746; doi:10.1261/rna.063479.117)

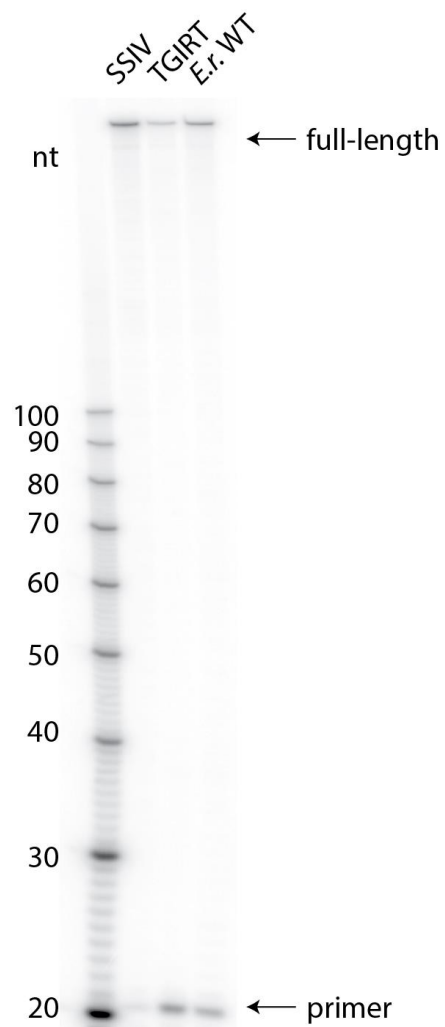

**Supplementary figure 1 Multi-turnover RT reaction using RepA D3 as template. SSIV: Superscript IV.**

Supplement: Supplemental Material [file supp_063479.117_Supplemental_Fig_S1.pdf]
