# Supplementary material for: An ultraprocessive, accurate reverse transcriptase encoded by a metazoan group II intron
Source: RNA. 2018 Feb;24(2):183–95. doi: 10.1261/rna.063479.117 (PMC5769746; doi:10.1261/rna.063479.117)
Supplement: Supplemental Material [file supp_063479.117_Supplemental_Fig_S2.pdf]

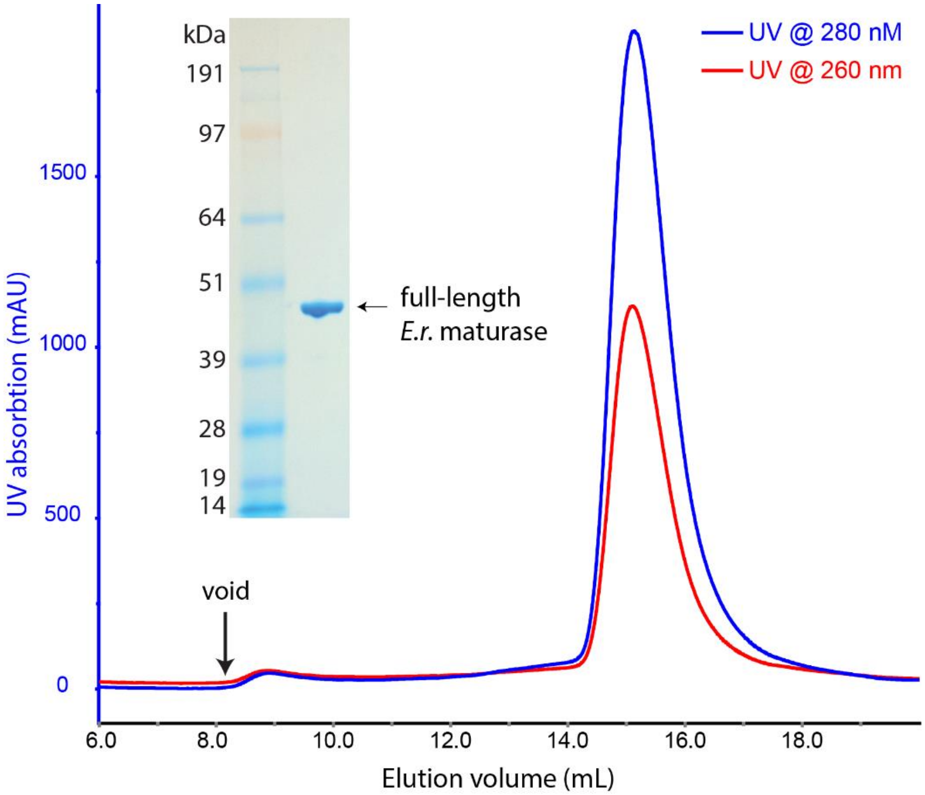

Supplementary figure 2 Chemical and conformational homogeneity of full-length *E.r.* maturase purified from *E.coli*.. The elution profile from Superdex S200 gel-filtration column (10/300 GL, GE Healthcare) suggests the almost all purified full-length *E.r.* maturase exist as monodispersed species. SDS-PAGE stained by Comassie suggests that purified *E.r.* maturase has high chemical purity.
