# Supplementary material for: An ultraprocessive, accurate reverse transcriptase encoded by a metazoan group II intron
Source: RNA. 2018 Feb;24(2):183–95. doi: 10.1261/rna.063479.117 (PMC5769746; doi:10.1261/rna.063479.117)
Supplement: Supplemental Material [file supp_063479.117_Supplemental_Table_S1.pdf]

RNA template	Annealing position	Sequence
RepA D1	387	5’ ACCATATTTCCATCCACCAAGCGC 3’
RepA D3	1630	5’ TAATAGGTGAGGTTTCAATG 3’
HCV genome	4940 (F)	5’ GTCTCCGCTGGTGTGAG 3’
HCV genome	5069 (E)	5’ GCTTGCTTTGTTTGGGAGAG 3’
HCV genome	5912 (D)	5’ AATGCCCGCACCATATCC 3’
HCV genome	8051 (C)	5’ GGAATTGGTGTTTGTGGGTC 3’
HCV genome	8953 (B)	5’ TTGGACCATGAGAATGGAGAAG 3’
HCV genome	9461 (A)	5’ AAGGAACAGTTAGCTATGGAGTG 3’

Supplementary Table 1 Primer sequences used for RT assays. Annealing position is the nucleotide number on RNA template that anneals to the very 5’ end of the primers.
