# Supplementary material for: An ultraprocessive, accurate reverse transcriptase encoded by a metazoan group II intron
Source: RNA. 2018 Feb;24(2):183–95. doi: 10.1261/rna.063479.117 (PMC5769746; doi:10.1261/rna.063479.117)
Supplement: Supplemental Material [file supp_063479.117_Supplemental_Table_S2.pdf]

	RT
2nd strand	CTACACGACGCTCTTCCGATCTCTGTNNNNNNNNNNNNNNNGATTATAGGACATTTAGGTCGTAC
RT	CAGACGTGTGCTCTTCCGATCGGTANNNNNNNNNNNNNNNACATTTCTAACTGGAAGTCAAGC
	PCR amplification
forward	CTACACGACGCTCTTCCGATCT
reverse	CAGACGTGTGCTCTTCCGATC
	Sequencing adaptors
index2	CAAGCAGAAGACGGCATACGAGATACATCGGTGACTGGAGTTCAGACGTGTGCTCTTCCGATCT
index4	CAAGCAGAAGACGGCATACGAGATTGGTCAGTGACTGGAGTTCAGACGTGTGCTCTTCCGATCT
index5	CAAGCAGAAGACGGCATACGAGATACAGTGGTGACTGGAGTTCAGACGTGTGCTCTTCCGATCT
index7	CAAGCAGAAGACGGCATACGAGATCAGATCGTGACTGGAGTTCAGACGTGTGCTCTTCCGATCT
universal	AATGATACGGCGACCACCGAGATCTACACTCTTTCCCTACACGACGCTCTTCCGATC

Supplementary Table 2 Primer sequences used for error rate determination. N indicates random nucleotide, and the string of 15 Ns is the product barcode (UMI). Blue color indicates condition barcode. Green and yellow regions indicate complementary sequences for PCR amplification. Red regions indicate Illumina index.
